# Inappropriate costimulation and aberrant DNA methylation as therapeutic targets in angioimmunoblastic T-cell lymphoma

**DOI:** 10.1186/s40364-017-0085-8

**Published:** 2017-02-08

**Authors:** Mathijs Willemsen, Harry C. Schouten

**Affiliations:** Department of Internal Medicine, Division of Hematology, Maastricht University Medical Centre, PO Box 5800, 6202 AZ Maastricht, The Netherlands

**Keywords:** Angioimmunoblastic T-cell lymphoma, Multistep tumorigenesis, Costimulation, DNA methylation, Molecular pharmacology

## Abstract

Angioimmunoblastic T-cell lymphoma (AITL) is one of the most common subtypes of peripheral T-cell lymphoma. Advances in understanding the mutational landscape of AITL have not resulted in improved prognosis nor consensus regarding optimal first-line and second-line treatment.

The recently proposed multistep tumorigenesis model for AITL provides a theoretical framework of AITL oncogenesis. In this model, early mutations in epigenetic modifiers interact with late cooperative mutations to enable malignant transformation. Frequent mutations in epigenetic modifiers suggest that aberrant DNA methylation contributes to AITL oncogenesis. Several research groups have reported findings suggesting that inappropriate costimulation acts as a late cooperative mutation. Drugs targeting inappropriate costimulation have already been approved for the treatment of several malignancies or autoimmune diseases. Additionally, aberrant DNA methylation was recently shown to potentiate inappropriate costimulation in a subset of AITL cases. Therefore, drugs targeting inappropriate costimulation and hypomethylating agents might have synergistic effects. Both offer promising new therapeutic options in AITL treatment.

This commentary summarizes the main findings on aberrant DNA methylation and inappropriate costimulation in AITL and proposes several already approved drugs for AITL treatment. Hopefully, these will contribute to improving the still dismal prognosis of AITL patients.

## Background

Peripheral T-cell lymphoma (PTCL) constitutes a heterogeneous group of non-Hodgkin lymphomas generally characterized by an aggressive clinical course and poor prognosis [1]. Angioimmunoblastic T-cell lymphoma (AITL) is one of the most common subtypes of PTCL accounting for 15–20% of all cases [2]. Advances in understanding the mutational landscape of AITL have not resulted in improved prognosis over the last two decades (5-year overall survival rate 25–41%), nor consensus regarding optimal first-line and second-line treatment [3]. Recently, a multistep tumorigenesis model for AITL was proposed [4]. In this model, early mutations in epigenetic modifiers interact with late cooperative mutations to enable malignant transformation. Frequent mutations in epigenetic modifiers suggest aberrant DNA methylation is involved in AITL oncogenesis. Several research groups have reported findings suggesting inappropriate costimulation acts as a late cooperative mutation in this model, thereby significantly contributing to AITL oncogenesis [5–8]. Drugs targeting inappropriate costimulation have already been approved for the treatment of several malignancies or autoimmune disorders. These drugs might be potent new therapeutic options in AITL treatment. Additionally, it was recently shown that aberrant DNA methylation is able to potentiate inappropriate costimulation [9]. These findings support the simultaneous use of hypomethylating agents and drugs targeting inappropriate costimulation in a subset of AITL cases. As these insights provide us with several new therapeutic options for AITL treatment, the efficacy of these already approved drugs should be evaluated in pre-clinical and clinical trials.

### Aberrant DNA methylation and multistep tumorigenesis in AITL

Varies sequencing studies have been conducted to investigate the mutational landscape of AITL. TET2, IDH2 and DNMT3A are mutated in 30–80%, 20–45% and 10–30% of investigated AITL cases, respectively [9–17]. Because all these genes encode epigenetic modifiers, it is suspected that aberrant DNA methylation somehow contributes to AITL oncogenesis.

Ten-Eleven Translocation 2 (TET2) is a Fe^2+^- and 2-oxoglutarate (2OG)-dependent dioxygenase involved in DNA demethylation. Most cytosine derivatives generated by TET2, especially 5-hydroxymethylcytosine resulting in DNA hydroxymethylation, are stable epigenetic marks [18]. Precisely how mutant TET2 contributes to AITL oncogenesis is unclear, but experimental data has shown that hematopoietic stem cells (HSCs) are particularly vulnerable to disruption of TET2 function resulting in increased self-renewal capacity and altered terminal differentiation [18]. Additionally, it was recently shown that intron1 of the BCL6 gene is frequently hypermethylated in mutant TET2 AITL cases [19]. B cell lymphoma 6 (Bcl6) is considered to be the lineage defining transcription factor for T follicular helper cells (T_FH_) [20]. Intron1 of the BCL6 gene has been identified as a silencer region, working in a methylation-sensitive manner in lymphoma cell lines [21]. Accordingly, Bcl6 was overexpressed in mutant TET2 AITL cases [19]. Similar results regarding BCL6 intron1 methylation and transcriptional upregulation have been reported in a TET2-knockdown mouse model which eventually develops T-cell lymphoma with T_FH_-like features [22]. Deregulated Bcl6 expression potentially alters T_FH_ differentiation and renders affected cells susceptible for malignant transformation after acquiring late cooperative mutations. Isocitrate dehydrogenase 2 (IDH2) is a potential regulator of TET2. Wildtype IDH2 catalyzes the two-step interconversion of isocitrate to 2OG. Mutant IDH2, in AITL restricted to R172 residue mutations, catalyzes the reduction of 2OG to (R)-2-hydroxyglutarate (2HG). 2HG has been identified as an oncometabolite potentially contributing to malignant transformation by competitively inhibiting 2OG-dependent enzymes. As these enzymes are involved in many cellular functions including DNA and histone modification, cellular energy metabolism and differentiation, further research is warranted to evaluate the oncogenic potential of downstream targets of mutant IDH2 [23]. A recent report further explored the role of mutant IDH2 in AITL. Tumor biopsies of nine untreated AITL patients only carried mutant IDH2 in the T-cell compartment, indicating that this mutation occurs in mature T-cells and not in HSCs [24]. Using a conditional knock-in mouse model the researchers investigated the effects of several IDH2 mutations in vivo. The R172 mutation, which is almost exclusively encountered in AITL, produced significantly more 2HG in the hematopoietic system than mutations at other residues. Additionally, the researchers found that only the R172 mutation impaired lymphoid development and inhibited TET2 function resulting in decreased DNA hydroxymethylation [24]. How this altered DNA hydroxymethylation pattern contributes to AITL oncogenesis is unknown. The greater 2HG generating capacity of the R172 mutations in lymphoid cells compared to mutations at other residues might explain why this mutation is exclusively found in AITL. Furthermore, the ability of the R172 mutation to inhibit TET2 function in vivo suggest a synergetic effect of both mutations in AITL oncogenesis. Wang et al. recently showed that mutant IDH2 is associated with hypermethylation of specific genes in AITL [9]. Among downregulated genes were negative regulators of T-cell signaling (PTPN7, SIT1 and DGKA). Protein tyrosine phosphatase non-receptor type 7 (PTPN7), also called hematopoietic PTP, is a mitogen-activated protein kinase (MAPK)-specific tyrosine phosphatase. PTPN7 inhibits T cell activation by dephosphorylating specific residues of MAPKs thereby preventing further downstream signaling along the Ras/Raf/MAPK/extracellular signal-regulated kinase (ERK) pathway [25]. SIT1 is a transmembrane adaptor protein involved in immune cell activation. SIT1 negatively regulates T cell activation by sequestering signaling molecules (e.g. Grb2) thereby preventing them from being recruited to the activated T cell receptor. This in turn prevents activation of downstream signaling pathways such as the Ras/Raf/MAPK/ERK pathway and thus T cell activation [26]. Diacylglycerol kinase alpha (DGKα) is a lipid kinase which converts diacylglycerol to phosphatidic acid, both important second messengers in several intracellular signaling pathways including Ras/Raf/MAPK/ERK and phosphatidylinositol 3 kinase (PI3K)/Akt/mammalian target of rapamycin (mTOR) signaling pathways. Whereas one might reason that DGKα inhibition would deplete second messengers and dampen signaling through these signaling pathways, evidence suggests the opposite is true. DGKα inhibition counterintuitively increases Ras activation and also T cell activation. It is therefore suspected that the in vivo effects of DGKα inhibition vary among different cell types [27]. As the evidence suggests that hypermethylation and subsequent downregulation of PTPN7, SIT1 and DGKα can potentially increase signaling through the Ras/Raf/MAPK/ERK pathway, it is possible that these epigenetic changes contribute to deregulated T cell activation and AITL oncogenesis. DNA methyltransferase 3A (DNMT3A) mediates de novo DNA methylation, a process which is relatively poorly understood. Experimental data has shown that loss of DNMT3A function results in impaired HSC differentiation and expansion of the HSC pool in the bone marrow. On methylation analysis, daughter cells exhibit global DNA hypomethylation and hypermethylation of specific CpG islands resulting in downregulation of early differentiation factors and upregulation of stem cell associated multipotency genes [28, 29]. It seems affected cells are skewed towards self-renewal instead of differentiation. This increased proliferative capacity likely contributes to AITL oncogenesis when affected cells acquire additional cooperative mutations. Interestingly, 70–100% of AITL patients with DNMT3A mutations also have TET2 mutations [9–17]. It was recently shown that co—occurrence of loss of function of TET2 and DNMT3A R882H mutation is able to drive the development of lymphoid malignancies, including AITL-like disease, in mice [30]. These data imply that DNMT3A and TET2 mutations have synergistic effects in AITL oncogenesis. As previously explained, both DNMT3A and TET2 potentially render HSCs susceptible for malignant transformation. Unfortunately, no DNA methylation analysis was performed on AITL-like mice. In addition to frequent mutations in epigenetic modifiers, RHOA is mutated in 50–70% of investigated AITL cases [9, 11, 12, 14]. Ras homologue family member A (Rhoa) is part of a subgroup of the Ras superfamily of GTP hydrolases. In the vast majority of AITL cases a missense mutation causes G17V substitution in the GTP/GDP-binding domain. Functional studies showed mutant Rhoa to act as a dominant-negative signaling protein, moderately increasing cell proliferation by increasing Akt phosphorylation [9, 11, 12, 14]. Recently, researchers generated a conditional knock-in mouse model which selectively induces mutant Rhoa expression in CD4^+^ T-cells [31]. E﻿xpression of mutant Rhoa resulted in spontaneous accumulation of T_FH_ with both increased expression of the Inducible T-cell CoStimulator (ICOS) receptor and increased ICOS-dependent signaling. Blocking ICOS signaling using an anti-ICOS antibody prevented mutant Rhoa-induced T_FH_ phenotype [31]. ICOS is a relatively new costimulatory molecule belonging to the immunoglobulin family of co-receptors, as does the relatively old costimulatory molecule CD28. ICOS is not constitutively expressed on naïve T-cells, rather its expression is induced by activation of T-cell receptor signaling and/or CD28 activation. ICOS signaling has shown to play a critical role in promoting the T_FH_ phenotype, likely via stabilization of Bcl6 [32]. It seems expression of mutant Rhoa in naïve T-cells induces T_FH_ phenotype via increased ICOS signaling. This mechanism might explain why this mutation is so frequently encountered in AITL.

Mutations in TET2 and DNMT3A are not only found in AITL but also in myeloid malignancies and even non-malignant HSCs. It is currently presumed that these mutations are acquired during an early stage of hematopoietic differentiation. Mutant HSCs cause clonal hematopoiesis and eventually give rise to preleukemic/prelymphoma stem cells with an increased risk for subsequent hematologic malignancies [33]. What kind of malignancy develops depends upon the pattern of acquired late cooperative mutations. It is currently suspected that mutations in RHOA and IDH2 are acquired at a later stage and contribute to AITL development from prelymphoma stem cells [4]. The first line of evidence validating the multistep tumorigenesis model for AITL has recently been published. The same research group that showed the T_FH_ phenotype-inducing capacities of mutant Rhoa in vivo, also explored the potentially synergistic effect of mutations in TET2 and RHOA. Expression of mutant Rhoa in HSCs derived from TET2-deficient mice resulted in the development of AITL. Neoplastic cells were heavily dependent upon ICOS signaling for growth and proliferation in vivo [31]. These are the first data to support the oncogenic potential of mutations in epigenetic modifiers interacting with cooperative mutations. Identifying additional cooperative mutations will increase our understanding of AITL oncogenesis and might offer new therapeutic options.

### T cell activation and evidence for inappropriate costimulation

T cell activation is determined by engagement of co-stimulatory and co-inhibitory molecules in combination with T cell receptor activation. As binding affinity of co-inhibitory molecules is generally higher compared to co-stimulatory molecules, T cell activation is mitigated when expression of ligands is low. This interplay functions to ensure appropriate T cell activation [34]. Engagement of co-stimulatory molecule CD28 by its ligands CD80 or CD86 initiates several intracellular signaling pathways via association of kinases and adaptor proteins to its cytoplasmic domains. These intracellular signaling pathways eventually culminate in activation of transcription factors (e.g. NF-AT, AP-1 and NF-κB) essential for T cell survival, proliferation and protein synthesis [35]. The PI3K/Akt/mTOR and Ras/Raf/MAPK/ERK intracellular signaling pathways are among the best studied signaling pathways downstream of CD28 [34].

Two research groups identified missense mutations in the gene encoding co-stimulatory molecule CD28 [5, 6]. Mutations at residue T195 and D124 were identified in 5-11% of investigated AITL cases. Using wildtype and mutant CD28-transfected cells both research groups explored the functional significance of these mutations. Binding affinity of CD28 D124V for CD86 was two times higher compared to wildtype CD28. The CD28 T195P mutant showed decreased binding affinity for PI3K regulatory heterodimer p85/p110, but increased binding affinity for adaptor proteins Grb2 and GRAP2. In CD28 T195P mutant-tranfected cells phosphorylation of downstream signaling proteins Akt, mTOR and ERK and proliferation rate were increased compared to wildtype CD28-transfected cells, either with or without stimulation. These findings are in line with the concept that mutant CD28 is constitutively active and promotes T cell proliferation. Both research groups confirmed enhanced NF-κB signaling in mutant CD28-tranfected cells compared to wildtype after stimulation [5, 6]. Gene expression profiling found increased T cell signal transduction signature in mutant CD28-positive AITL cases compared to wildtype CD28 cases [6]. Additionally, mutant CD28-positive AITL cases showed decreased overall survival compared to wildtype cases [6]. These finding are in accordance with a previous report showing decreased overall survival in AITL and PTCL not otherwise specified cases with activation of NF-κB signaling [36]. Yoo et al. reported the presence of a CTLA4-CD28 fusion gene in 58% of investigated AITL cases in an Asian cohort. [7]. The corresponding fusion protein consists of the extracellular and transmembrane domains of co-inhibitory molecule cytotoxic T-lymphocyte antigen-4 (CTLA-4) and the cytosolic domain of co-stimulatory molecule CD28. This suggests that the fusion protein can bind ligands with the affinity of CTLA-4 while mediating the T cell activating effects of CD28. Indeed, functional study of fusion gene-transfected cells showed increased phosphorylation of Akt and ERK and increased proliferation rate compared to CTLA-4 wildtype-transfected cells on stimulation [7]. The effect of CTLA4-CD28 fusion protein activation on NF-κB signaling or gene expression has not been assessed. Yoo et al. did however evaluate the mutational landscape in which the fusion gene occurred. The frequency of the CTLA4-CD28 fusion gene was 38% (11/29) in deep sequenced AITL cases. None of the fusion gene-positive AITL cases carried a mutation in CD28, whereas 22% (4/18) of fusion gene-negative AITL cases did carry a C28 mutation [7]. These data suggest that occurrence of the CTLA4-CD28 fusion gene and CD28 mutations are mutually exclusive. In summary, both CD28 mutations and CTLA4-CD28 fusion increases intracellular signaling downstream of CD28 via PI3K/Akt/mTOR and Ras/Raf/MAPK/ERK signaling pathways resulting in increased survival and proliferation. Together these in vitro studies provide strong evidence for inappropriate costimulation as potential driver of AITL oncogenesis. In the multistep tumorigenesis model for AITL, inappropriate costimulation would therefore classify as a cooperative mutation which potentially contributes to AITL development from prelymphoma stem cells.

A recently published study expanded upon these earlier findings indicating inappropriate costimulation as potential cooperative mutation in AITL oncogenesis. Vallois et al. showed that 50% (36/72) of investigated AITL cases had mutations in components of T cell receptor signaling or costimulatory pathways, other than RHOA [8]. Intriguingly, these mutations had a nearly mutually exclusive nature thereby confirming the findings by Yoo et al. [7]. CD28 was mutated in 11% (8/72) of investigated AITL cases. Additionally, several components of both the PI3K/Akt/mTOR and Ras/Raf/MAPK/ERK signaling pathways were mutated. The vast majority of mutations were classified as gain-of-function. Enrichment analysis of AITL cases with mutations in T cell receptor signaling or costimulatory pathways compared to AITL cases without such mutations showed significantly enriched molecular signatures reflecting activation of several signaling pathways including PI3K and NF-κB. These findings are in line with previous reports showing increased activation NF-κB signaling in mutant CD28 AITL cases [5, 6]. The nearly mutually exclusive nature of these mutations implies that a single gain-of-function mutation in T cell receptor signaling or costimulatory pathways, which are regarded as essential for T cell survival and are tightly regulated under physiological conditions, is sufficient to contribute to full blown malignant transformation in a subset of AITL cases. Together, these data underline inappropriate costimulation as cooperative mutation and significant contributor to AITL oncogenesis.

### Targeting inappropriate costimulation

**Fig. 1:**
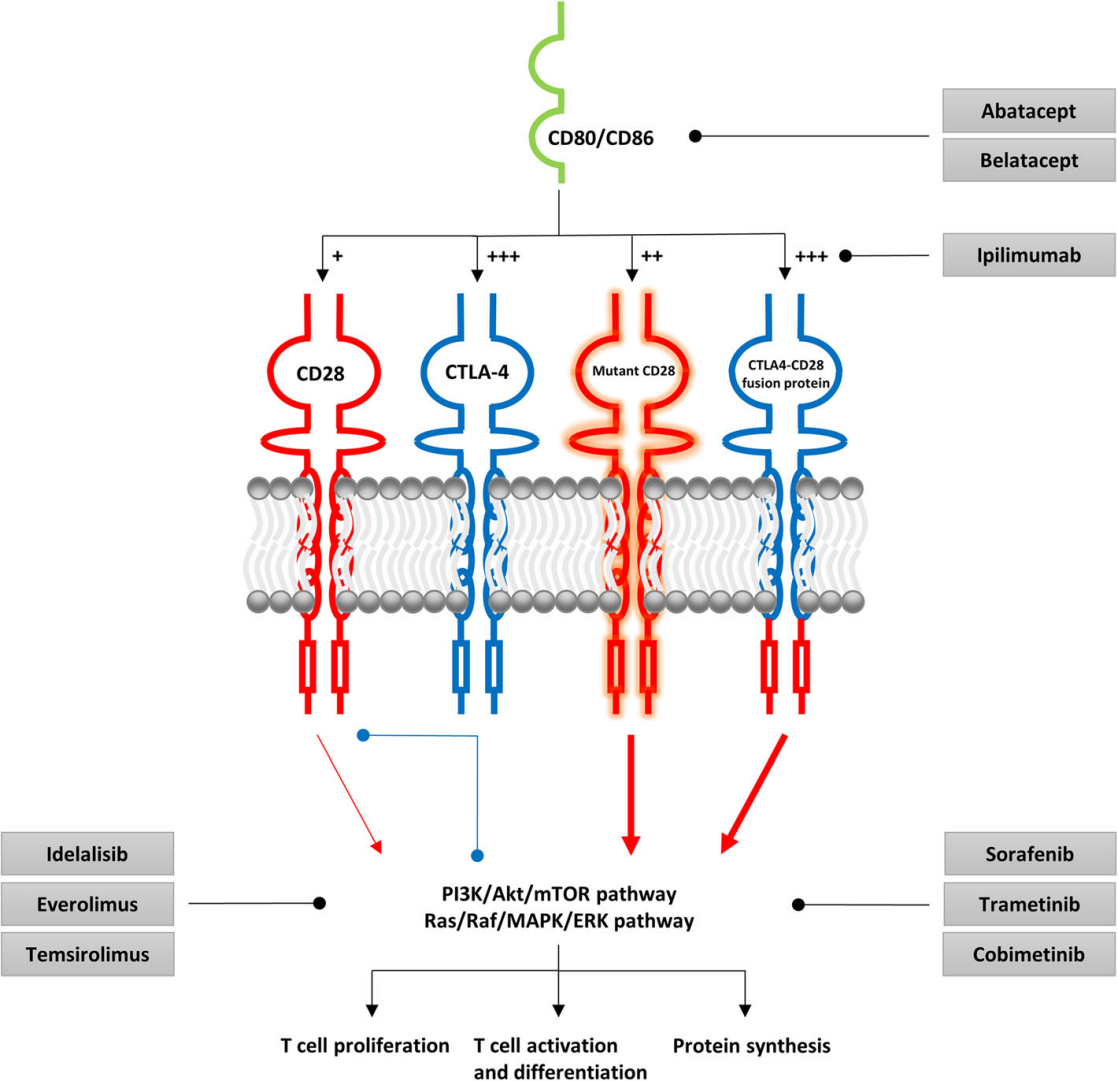
Potential inhibitors of inappropriate costimulation in AITL. CD28 is a costimulatory molecule essential for proper T cell activation. CD80/CD86 binding initiates several intracellular signaling pathways via association of kinases and adaptor proteins to its cytoplasmic domains. These in turn activate several intracellular signaling pathways (e.g. PI3K/Akt/mTOR and Ras/Raf/MAPK/ERK) resulting in proliferation, activation and differentiation and protein synthesis. CTLA-4 is a co-inhibitory molecule mitigating T cell activation. In general, CTLA-4 inhibits T cell activation by three mechanisms: competition for ligand binding, diminution of intracellular signaling protein recruitment and direct inhibition of downstream signaling pathways. Both mutant CD28 and the CTLA4-CD28 fusion protein mediate inappropriate costimulation potentially driving proliferation of malignant cells in AITL. Various already approved drugs can potentially block inappropriate costimulation by preventing ligand-coreceptor interaction or inhibiting the PI3K/Akt/mTOR and Ras/Raf/MAPK/ERK signaling pathways. Red arrows: activation, Blue dots: inhibition

Targeting inappropriate costimulation in AITL might prove an effective therapeutic approach. Two strategies could potentially be effective in blocking inappropriate costimulation: 1) preventing ligand-coreceptor interaction and 2) inhibition of intracellular signaling pathways downstream of co-stimulatory molecule CD28 (Fig. 1).

Various drugs targeting ligand-coreceptor interaction have already been approved. Ipilimumab (Yervoy; Bristol-Myers Squibb) is a CTLA-4-specific monoclonal antibody which can potentially be effective in CTLA4-CD28 fusion gene-positive AITL patients. Ipilimumab has already been approved for the treatment of malignant melanoma [37]. Abatacept (Orencia; Bristol-Myers Squibb) and belatacept (Nulojix; Bristol-Myers Squibb), both CTLA4-immunoglobulin fusion proteins, are able to bind coreceptor ligands CD80 and CD86, thereby preventing activation of mutant CD28 or the CTLA4-CD28 fusion protein. Abatacept is already approved for the treatment of rheumatoid arthritis, whereas belatacept is approved for prevention of kidney transplant rejection [38].

Various components of the PI3K/Akt/mTOR signaling pathway have been targeted by specific inhibitors. Idelalisib (Zydelig; Gilead Sciences) inhibits class I PI3K isoform p110δ, the main PI3K isoform expressed in leukocytes. Idelalisib is already approved for the treatment of certain hematological malignancies (e.g. follicular B-cell non-Hodgkin lymphoma). In vitro data has shown that p110δ is the main PI3K isoform responsible for PI3K activity at the immune synapse [39]. Because mutant CD28 has decreased binding affinity for PI3K regulatory heterodimer p85/p110, it remains to be seen if inhibiting p110δ has an anti-proliferative effect in mutant CD28 AITL cases. However, a previous report has shown that CD28 can enhance PI3K activity independently of its capacity to interact with the p85/p110 heterodimer [39]. Potentially mutant CD28 activates PI3K via other mechanisms. Therefore, combined targeting of CD28 and class I PI3K isoform p110δ might prove a potent therapeutic option in mutant CD28 AITL cases. Idelalisib as a single agent, or in combination with a CTLA-4 blocker, could also be effective in CTLA4-CD28 fusion gene-positive AITL cases. Furthermore, two mTOR inhibitors, everolimus (Afinitor; Novartis) and temsirolimus (Torisel; Pfizer), have been approved for the treatment of renal cell carcinoma. Temsirolimus has also been approved for the treatment of relapsed/refractory (R/R) mantle cell lymphoma. Both agents initially only inhibit the mTORC1 protein complex and not the mTORC2 complex, whereas CD28 costimulation has shown to induce both mTORC1- and mTORC2-dependent signaling [40]. In vitro data has shown that prolonged exposure to rapamycin impairs mTORC2 complex assembly in certain cell types, including Jurkat and BJAB cells [41]. It is likely that everolimus and temsirolimus, both rapamycin analogs, exert the same cell-dependent effect on mTORC2. As both mutant CD28 and CTLA4-CD28 fusion gene-transfected cells showed increased phosphorylation of mTOR, prolonged exposure to mTOR inhibitors might prove a potent therapeutic option in mutant CD28 and CTLA4-CD28 fusion gene-positive AITL cases. A recent phase II clinical trial on the treatment of newly diagnosed PTCL patients with everolimus and CHOP showed complete response in all included AITL cases (3/3) [42]. The mutational landscape of included AITL patients is unknown. It is suspected that mTOR-dependent signaling plays a role in T_FH_ differentiation which could also explain the efficacy of everolimus in AITL [43].

Most of the approved Ras/Raf/MAPK/ERK signaling pathway inhibitors target frequently mutated components. Sorafenib (Nexavar; Bayer), a multikinase inhibitor (including Raf), and trametinib (Mekinist; GlaxoSmithKline) and cobimetinib (Cotellic; Genentech/Exelixis), both MEK inhibitors, are currently the only approved non-BRAF mutant-selective Ras/Raf/MAPK/ERK signaling pathway inhibitors [44]. Sorafenib is approved for the treatment of renal cell carcinoma, hepatocellular carcinoma and thyroid carcinoma, whereas trametinib and cobimetinib are approved for the treatment of malignant melanoma. It is likely that activation of mutant CD28 results in increased Raf and MEK phosphorylation due to increased binding affinity for upstream Grb2 and GRAP2 and increased phosphorylation of downstream ERK [5, 6]. Activation of CTLA4-CD28 fusion protein also resulted in increased ERK phosphorylation [7]. These agents might therefore be a potent therapeutic option in mutant CD28 and CTLA4-CD28 fusion gene-positive AITL cases.

### Combined targeting of inappropriate costimulation and aberrant DNA methylation

TET2 and DNMT3A are among the most frequently mutated genes in AITL [9–17]. The multistep tumorigenesis model for AITL focuses on the interaction between early mutations in these epigenetic modifiers and late cooperative mutations enabling malignant transformation [4]. As previously explained, a growing body of evidence suggests that aberrant DNA methylation predisposes to malignant transformation. Despite availability and experience with hypomethylating agents in other hematological diseases, targeting aberrant DNA methylation is not part of current AITL treatment.

Azacitidine (Vidaza; Celegene) and decitabine (Dacogen; Otsuka) are currently the only two approved hypomethylating agents for the treatment of myelodysplastic syndromes and acute myeloid leukemia [45]. Azacitidine and decitabine are nucleoside analogues that incorporate into newly synthesized DNA and RNA strands and irreversibly inhibit DNMTs. Both drugs primarily inhibit DNMT1, which is responsible for maintaining DNA methylation patterns after DNA replication [46]. Loss of DNMT1 function results in DNA hypomethylation and potentially prevents the aberrant DNA methylation pattern in AITL from being epigenetically inherited by daughter cells. DNMT3A is only inhibited at higher doses of azacitidine in vitro [46]. Two case reports have shown promising response of R/R AITL patients when treated with azacitidine [47, 48]. Both patients carried TET2 mutations and achieved complete response. Additionally, a recent report on 12 R/R AITL patients being treated with azacitidine, including both previously published case reports, showed an overall response and complete response rate of 75 and 42%, respectively [49]. Only two of the initial responders experienced disease progression after 86 and 499 days. Intriguingly, all initial responders with available TET2 mutational status had TET2 mutations (8/8). Additional DNA sequencing studies are undergoing to further investigate the mutational landscape of included AITL cases. Further prospective data are needed to confirm these findings, but it is clear that targeting aberrant DNA methylation in AITL is a promising therapeutic option.

Combined targeting of inappropriate costimulation and aberrant DNA methylation may prove to increase therapeutic efficacy in some AITL cases. Several negative regulators of T cell receptor signaling (PTPN7, SIT1 and DKGA) have shown to be hypermethylated and downregulated in mutant IDH2 AITL cases [9]. As previously explained, downregulation of these genes could potentially lead to increased signaling through the Ras/Raf/MAPK/ERK pathway downstream of mutant CD28 or the CTLA4-CD28 fusion protein, thereby potentiating inappropriate costimulation and contributing to AITL oncogenesis [25–27]. The various targets of negative regulators of T cell receptor signaling are located at multiple levels downstream of mutant CD28 and the CTLA4-CD28 fusion protein. This suggests that only targeting inappropriate costimulation at a single level by using the proposed drugs is insufficient to completely abolish inappropriate costimulation. We hypothesize that the use of hypomethylating agents in mutant IDH2 AITL cases induces DNA hypomethylation, thereby restoring expression of the negative regulators of T cell receptor signaling and subsequently abolishing their potentiating effect on inappropriate costimulation. If inappropriate costimulation is simultaneously targeted, this potentially eradicates signaling through costimulatory pathways. The recent findings by Vallois et al., showing that 50% of investigated AITL cases had mutations in components of T cell receptor signaling or costimulatory pathways, highlight the importance of deregulated signaling through costimulatory pathways, including the Ras/Raf/MAPK/ERK pathway, in AITL oncogenesis [8]. Together with the fact that aberrant DNA methylation contributes to AITL oncogenesis, these findings provide a strong biological and theoretical rationale for the simultaneous use of hypomethylating agents and drugs targeting inappropriate costimulation in mutant IDH2 AITL cases with mutations in costimulatory pathways.

## Conclusion

Advances in understanding the mutational landscape of AITL have not resulted in improved prognosis nor consensus regarding optimal first-line and second-line treatment. However, these findings have resulted in a multistep tumorigenesis model for AITL which provides us with a theoretical framework for AITL oncogenesis. Several research groups have identified mutations in costimulatory pathways resulting in inappropriate costimulation, potentially contributing to AITL oncogenesis. We propose that inappropriate costimulation can be regarded as a new cooperative mutation for AITL development from prelymphoma stem cells. This insight opens new therapeutic options for AITL treatment. We have proposed several candidate drugs with the potential to block inappropriate costimulation which have already been approved for the treatment of other malignancies or autoimmune disorders. Furthermore, we provided an overview of the current knowledge regarding aberrant DNA methylation in AITL. Hypomethylating agents are already being used to treat other hematological disorders and first prospective data for the use of hypomethylating agents in AITL treatment are promising. Additionally, studies have shown that aberrant DNA methylation can increase signaling through costimulatory pathways and therefore potentiate inappropriate costimulation. Together with the fact that aberrant DNA methylation contributes to AITL oncogenesis, these findings provide a biological and theoretical rationale for the simultaneous use of hypomethylating agents and drugs targeting inappropriate costimulation in a subset of AITL cases. Pre-clinical studies and clinical trials should be readily conducted to investigate the efficacy of these already approved drugs and their potential synergistic effect. Hopefully, these insights will contribute to improving the still dismal prognosis of AITL patients.

## Abbreviations

2HG: (R)-2-hydroxyglutarate
2OG: 2-Oxoglutarate
AITL: Angioimmunoblastic T-cell lymphoma
Bcl6: B cell lymphoma 6
CTLA-4: Cytotoxic T-lymphocyte antigen-4
DGKα: Diacylglycerol kinase alpha
DNMT3A: DNA methyltransferase 3A
HSCs: Hematopoietic stem cells
IDH2: Isocitrate dehydrogenase 2
MAPK: Mitogen-activated protein kinase
PI3K/Akt/mTOR: Phosphatidylinositol 3 kinase/Akt/mammalian target of rapamycin
PTCL: Peripheral T-cell lymphoma
PTPN7: Protein tyrosine phosphatase non-receptor type 7
R/R: Relapsed/refractory
Ras/Raf/MAPK/ERK: Ras/Raf/MAPK/extracellular signal-regulated kinase
RHOA: Ras homologue family member A
TET2: Ten-eleven translocation 2
T_FH_: T follicular helper cell
